# Rehabilitation of Patients With Acute Ischemic Stroke Who Required Assistance Before Hospitalization Contributes to Improvement in Activities of Daily Living: A Nationwide Database Cohort Study

**DOI:** 10.1016/j.arrct.2022.100224

**Published:** 2022-08-05

**Authors:** Takuaki Tani, Shinobu Imai, Kiyohide Fushimi

**Affiliations:** a Tokyo Medical and Dental University Graduate School of Medical and Dental Sciences, Tokyo, Japan; b Clinical Research Center National Hospital Organization, Tokyo, Japan; c Tokyo University of Pharmacy and Life Sciences, Tokyo, Japan

**Keywords:** Activities of daily living, Delivery of health care, Hospitals, Ischemic stroke, Outcome assessment, health care, Rehabilitation

## Abstract

Objective: To assess the effect of early implementation of and longer daily duration of rehabilitation on patients with acute ischemic stroke who require assistance with activities of daily living (ADL) before hospital admission.

Design: Nationwide, cohort, observational study from April 2018 to March 2019.

Setting: Acute care hospitals in Japan.

Participants: The Japanese national Diagnosis Procedure Combination database was searched for the period between April 2018 and March 2019. Of the 330,672 patients with ischemic strokes identified, 53,523 met the inclusion criteria of being older than 20 years, having a prehospital modified Rankin Scale score of 3, 4, or 5, and having undergone rehabilitation (N=53,523).

Interventions: Not applicable.

Main Outcome Measures: Improvement in ADL from admission to discharge using the Barthel Index. The effects of the following 3 rehabilitation variables on ADL improvement were evaluated: (1) average daily duration of rehabilitation; (2) rehabilitation started within 3 days after admission (early rehabilitation); and (3) rehabilitation started 1 day after admission (very early rehabilitation).

Results: Early rehabilitation was significantly associated with improvements in ADL (odds ratio, 1.19; 95% confidence interval, 1.10-1.28; *P*≤.001). A longer duration of rehabilitation was also significantly associated with ADL improvement (≥2.0 hours: odds ratio, 2.49; 95% confidence interval, 2.26-2.75; *P*≤.001) compared with a ≤1 hour of rehabilitation (1.1-2.0 hours: odds ratio, 1.35; 95% confidence interval, 1.29-1.42; *P*≤.001).

Conclusions: Early implementation of rehabilitation and a longer duration of rehabilitation per day improved the ADL of patients who required assistance before the onset of cerebral infarction.

## HIGHLIGHTS

• We conducted a nationwide observational study in hospitalized patients with acute stroke.
• We studied the value of rehabilitation in those requiring assistance prehospitalization.
• Early implementation and longer duration of rehabilitation per day improved activities of daily living.
• Indications for early rehabilitation should be based on background factors.

Because ischemic stroke causes physical dysfunction, rehabilitation is important to improve functional impairment after the onset of stroke.^1^ As the world's population ages, many patients have disabilities even before the onset of stroke.^2^^,^^3^ Previous studies have revealed the effectiveness of early rehabilitation and the necessity of a certain duration of rehabilitation for patients with acute ischemic stroke.^4^, ^5^, ^6^, ^7^ Accordingly, medical guidelines contain recommendations about the timing and duration of rehabilitation.^8^ However, these previous studies were limited to patients who were living independently before hospitalization, and the effects on patients who required assistance before hospitalization have not been clarified.

High physical status and activity before hospitalization reportedly contribute to the recovery of activities of daily living (ADL) after hospitalization.^9^ A study showed that a prehospital exercise habit of 30 minutes or more per day was significantly associated with functional recovery after stroke compared with no exercise habit.^10^ Thus, physical activity before the onset of illness and rehabilitation after stroke influence functional recovery after hospitalization; however, rehabilitation in the acute phase for patients who required assistance before the onset of stroke has not been sufficiently investigated.

We hypothesized that the effect of early rehabilitation on patients with acute stroke who needed assistance prior to admission might be smaller than on those who are independent before admission. The purpose of this study was, therefore, to investigate the effectiveness of rehabilitation for ADL improvement in patients with acute ischemic stroke who required assistance with ADL before admission.

## Methods

### Study design

This was a nationwide, observational study based in Japan from April 2018 to March 2019 in a cohort of hospitalized patients.

### Patient selection

We extracted the data of inpatients who were diagnosed with cerebral infarction at the time of admission (April 2018 to March 2019) from the Japanese national Diagnosis Procedure Combination (DPC) database. The *International Statistical Classification of Diseases, Tenth Revision* codes were used for the identification of cerebral infarction (I63). Patients 20 years or older (Japanese adult standard), those with a prehospital modified Rankin Scale (mRS) score of 3, 4, or 5 (indication criterion; a score of ≥3 was defined as those who required assistance before admission), and those who underwent rehabilitation were included in the study.^11^ We excluded patients who stayed in the hospital for over 180 days and those who died during hospitalization. Rehabilitation costs were not reimbursed beyond 180 days for the eligible patients in our study.

### Data source and variables

DPC is a national acute care inpatient database in Japan; it uses a fixed payment system per hospitalized day. This database covers 55% of hospitalized patients in Japan. The DPC system is mandatory for all university hospitals.^12^ The 45% of acute care hospitals not covered by this database are community hospitals that do not use the DPC system. In this study, data approved for a study of 1262 hospitals were used. The data include age, sex, weight, height, use of emergency transportation, complications, comorbidities, medical procedures, length of hospital stay (LOS), mRS, the 20-point scale of Barthel Index (BI),^13^ and Japan Coma Scale (JCS).^14^ Medical procedure information includes daily records of types of surgery, medical prescriptions, and rehabilitation implementation data. The main diagnosis complications and comorbidities were coded using the *International Statistical Classification of Diseases, Tenth Revision*.

Age was divided into 5 groups: 59 or younger, 60-69, 70-79, 80-89, and 90 years or older, because the incidence of stroke increases after the age of 50 years and because we analyzed age distribution and decided to stratify it into 5 groups. We calculated the body mass index at admission from the recorded height and weight, assigned based on modified World Health Organization classifications.^15^ The Charlson Comorbidity Index (CCI) was calculated using Quan's protocol.^16^ For the calculation of the CCI, the patients’ comorbidities were classified into 17 categories. Each disease was assigned a weighted score of 1, 2, 3, or 6, depending on the risk of death associated with that disease. The individual scores were summed to calculate the total CCI score; the total CCI score was divided into 4 groups: 0, 1, 2, and ≥3.^16^^,^^17^ The JCS score at admission was used as a consciousness scale. A JCS code 0 denotes a patient without impaired consciousness, codes 1-3 denote a patient who is awake without stimuli, codes 10-30 denote a patient who can be roused by some stimuli, and codes 100-300 denote a patient in a coma. This study used 4 categories for the JCS: 1-3, 10-30, 100-300, and 0. We checked the distribution of the LOS and stratified the LOS into 2-week periods for up to 180 days. Discharge destinations were categorized as home, transfer, institutional, and others with unknown destination. Treatments extracted from the database were nasogastric tube, percutaneous endoscopic gastrostomy, nutritional intake through a gastrostomy, tissue plasminogen activator, edaravone, mechanical thrombectomy categorized by the Japanese surgical code K178-4, and admission to the stroke care unit.

### Rehabilitation

The average daily duration of rehabilitation, calculated as the total duration of rehabilitation during hospitalization divided by the number of dates of rehabilitation performed, was categorized into 3 groups: ≤1.0 hours, 1.1-2.0 hours, and ≥2.1 hours. Early rehabilitation was defined when rehabilitation was started within 3 days of admission, and the following categorical variables were created: early rehabilitation and usual rehabilitation early. Very early rehabilitation was defined as when rehabilitation was started within 1 day of admission.

### Outcome measurement

The primary outcome was ADL improvement from admission to discharge using the BI. We subtracted the BI score at discharge from that at admission and created categorical variables. Based on a previous study, the minimal clinically important difference of the BI was defined as 1.85 points.^18^ The clinical effect difference refers to the difference between the magnitude of the change in ADL function in response to exposure and the perceived improvement in ADL by the patient. If the BI of the ADL was >1.85, it was defined as ADL improvement.

### Statistical methods

The basic characteristics were described, and categorical variables are expressed as the number of participants and percentage. Continuous variables are expressed as mean.

We tested whether there were differences in the BI, LOS, and discharge destination in the very early and early rehabilitation groups and between the duration of rehabilitation per day groups. The outcome of this study was the difference between ADL at admission and discharge because ADL is affected by LOS and discharge destination.

For the main analysis, multivariable logistic regression models were constructed for early rehabilitation, duration of rehabilitation per day, and independent variables that were related to the outcome in prior studies.^6^^,^^7^ All variables were cross-tabulated to assess for multicollinearity in case 2 variables correlated by >0.25 with each other, which was acceptable for the subsequent analysis.^19^ Additionally, patients were divided into 3 groups: mRS3, mRS4, and mRS5; we analyzed the effect of early rehabilitation, very early rehabilitation, and the duration of rehabilitation for ADL improvement. For the sensitivity analysis, starting rehabilitation within 1 day of admission was defined as very early rehabilitation. Then, multivariable logistic regression models were constructed to compare very early rehabilitation with other covariates as the main analysis of ADL improvement.

In the regression model, missing data of ADL were assigned using multiple imputation with the chained equations method.^20^ Using multiple imputation, we created 100 data sets for estimates and SEs of the multivariable logistic regression models. All missing data of covariates were included for analysis as “NA” except for missing BI data for the outcome. Statistical analyses were performed using R statistical software version 4.2.0.^a^

### Ethical approval

Study approval was obtained from the institutional review board of the Tokyo Medical and Dental University (M2000-788-27). Because of the anonymous nature of the data, the requirement for informed consent was waived.

## Results

**Fig 1:**
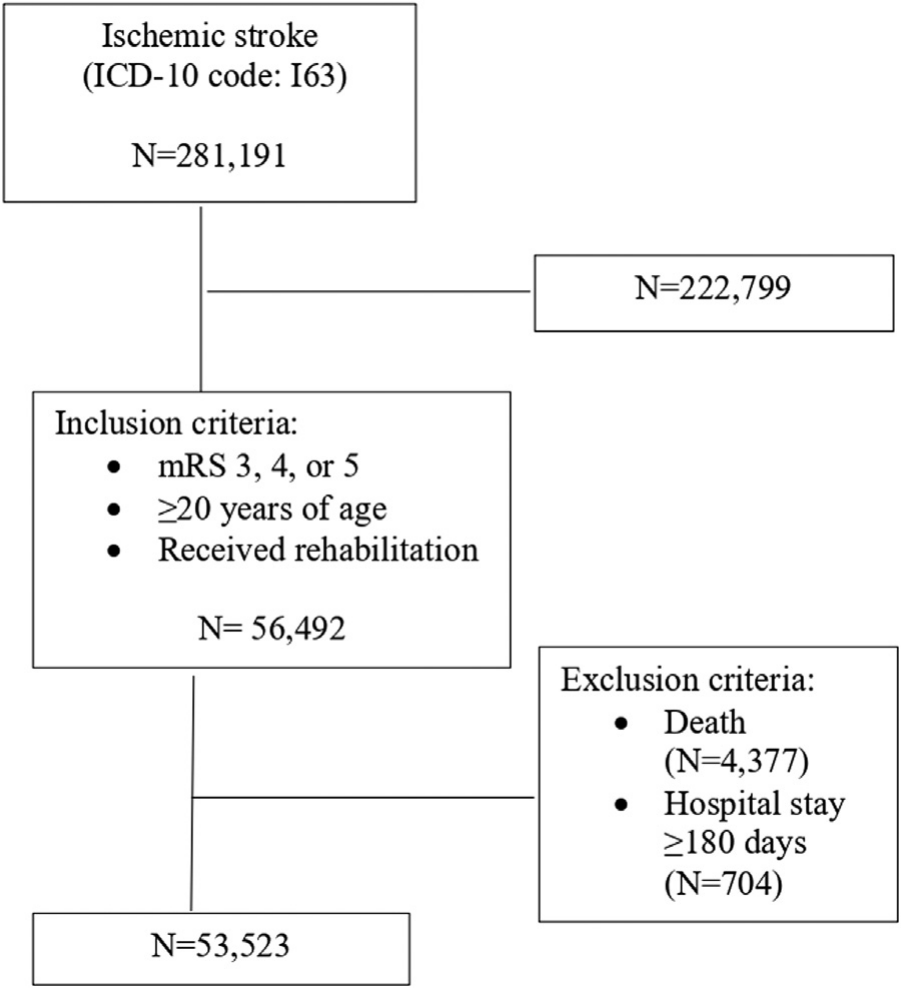
Flowchart of the study. Abbreviation: ICD-10, *International Statistical Classification of Diseases, Tenth Revision.*

A total of 330,672 ischemic stroke cases were extracted from the database; 53,523 cases were eligible for analysis considering the eligibility criteria (fig 1). Of these, 11,169 cases had missing data (20.8% of the sample size); therefore, the missing data were in the range that could be imputed (supplemental table S1).^21^

Table 1 shows the baseline characteristics of the patients. The highest incidence of stroke was in patients aged 80-89 years (n=22,857 [42.7%]), and the proportion of women was higher (n=29,362 [54%]); mRS score before admission was the highest in mRS3 (n=22,870 [42.7%]). The proportion of tissue plasminogen activator (tPA) performed was 4.8%.

**Table 1:** Baseline characteristics

Characteristics		Total (N=53,523)
Age (y), n (%)	≤59	1854 (3.5)
	60-69	4571 (8.5)
	70-79	12,174 (22.7)
	80-89	22,852 (42.7)
	≥90	12,072 (22.6)
Sex (female), n (%)		29,362 (54.9)
BMI, n (%)	≤18.5	9771 (18.3)
	18.5-24.9	30,698 (57.4)
	25-29.9	7438 (13.9)
	30-34.9	1060 (2.0)
	≥35	2158 (4.0)
	NA	2398 (4.5)
CCI, n (%)	0	16,667 (31.1)
	1	17,906 (33.5)
	2	11,217 (21.0)
	≥3	7733 (14.4)
mRS score	3	22,870 (42.7)
	4	22,686 (42.4)
	5	7967 (14.9)
JCS at admission, n (%)	0	16,927 (31.6)
	1	27,109 (50.6)
	2	6768 (12.6)
	3	2719 (5.1)
Emergency transportation, n (%)	0	24,486 (45.7)
	1	29,015 (54.2)
	NA	22 (0.0)
Hospital readmission, n (%)		2778 (5.2)
Percutaneous endoscopic gastrostomy, n (%)		1271 (2.4)
Nasogastric tube, n (%)		10,823 (20.2)
Edaravone, n (%)		26851 (50.2)
Tissue plasminogen activator, n (%)		2582 (4.8)
Mechanical thrombectomy, n (%)		1863 (3.5)
Stroke care unit, n (%)		7695 (14.4)

NOTE. BMI calculated as weight in kilograms divided by height in meters squared.

Abbreviation: NA, not applicable.

**Fig 2:**
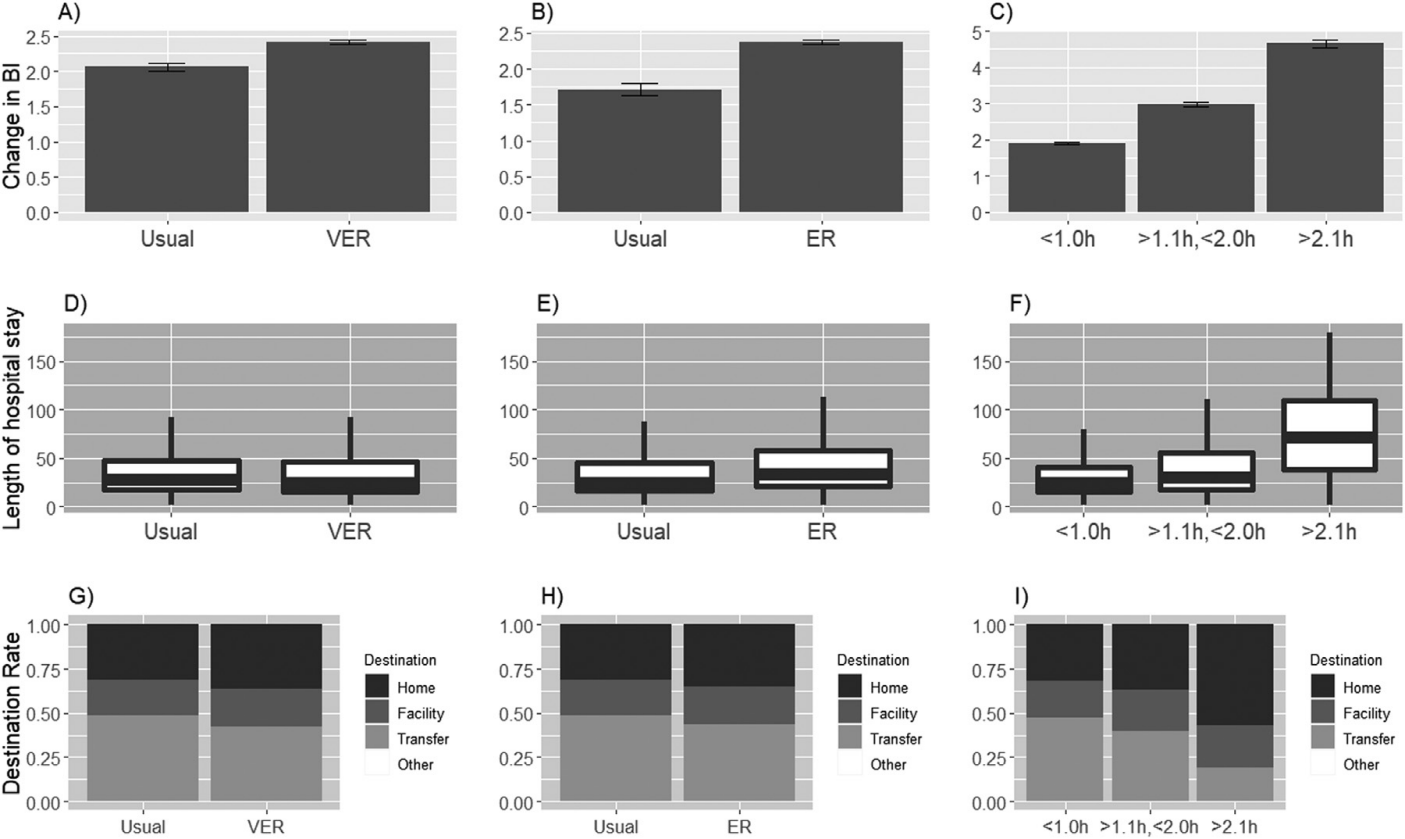
Outcomes and discharge variables for each rehabilitation group. (A) Very early rehabilitation vs usual rehabilitation for crude BI values; (B) early rehabilitation vs usual rehabilitation for crude BI values; (C) rehabilitation duration per day for crude BI values; (D) very early rehabilitation vs usual rehabilitation for LOS; (E) early rehabilitation vs usual rehabilitation for LOS; (F) rehabilitation duration per day for LOS; (G) very early rehabilitation vs usual rehabilitation for discharge destination; (H) early rehabilitation vs usual rehabilitation for discharge destination; (I) rehabilitation duration per day for discharge destination, Abbreviations: ER, early rehabilitation; VER, very early rehabilitation.

Figure 2 and supplemental tables S2-S4 show the associations between very early rehabilitation, early rehabilitation, the duration of rehabilitation per day, and BI value at discharge. Crude BI values were significantly higher in the very early and early rehabilitation groups than in the usual rehabilitation groups (very early rehabilitation [2.4±5.5] vs usual rehabilitation [2.1±6.0], *P*<.001; early rehabilitation [2.4±5.6] vs usual rehabilitation [1.7±6.1]; *P*<.001). Crude BI values decreased significantly as rehabilitation duration per day increased (≤1.0 hours [1.9±5.7] vs 1.1-2.0 hours [3±5.6] vs ≥2.1 hours [4.6±5.0]; *P*<.001). The implementation of very early rehabilitation and early rehabilitation occurred at a higher rate than usual rehabilitation (very early rehabilitation [66.4%] and usual rehabilitation [33.7%]; early rehabilitation [88.0%] and usual rehabilitation [12.0%]). The most common duration of rehabilitation per day was ≤1 hour (≤1.0 hours [73.3%], 1.1-2.0 hours [21.4%], ≥2.1 hours [5.2%]). The median LOS was significantly longer in the usual rehabilitation groups, and it was increased as the duration of rehabilitation per day increased (*P*<.001). The discharge destination to the facility was a significantly higher rate in usual rehabilitation than very early and early rehabilitation (*P*<.001). The discharge destination to home was a significantly higher rate in the duration of rehabilitation per day in the >2.1 hours group (*P*<.001).

Table 2 shows the relationship between rehabilitation and ADL improvement using multivariable logistic regression analysis with multiple imputation. Early rehabilitation was significantly associated with improvements in ADL (*P*≤.001), and a longer duration of rehabilitation per day was also significantly associated with ADL improvement compared with a duration of rehabilitation ≤1 hour (1.1-2.0 hours, *P*≤.001; ≥2.0 hours, *P*≤.001).

**Table 2:** Relationship between rehabilitation and ADL improvement using multivariable logistic regression analysis with multiple imputation

Variable		Odds Ratio	95% CI	*P* Value
Early rehabilitation	Usual	Reference		
	Early	1.19	1.10-1.28	<.001
Duration of rehabilitation per day (h)	≤1	Reference		
	1.1-2.0	1.35	1.29-1.42	<.001
	≥3.1	2.49	2.26-2.75	<.001
Age (y)	≤59	Reference		
	60‐69	0.90	0.79-1.03	.133
	70-79	0.79	0.70-0.88	<.001
	80-89	0.64	0.57-0.72	<.001
	≥90	0.49	0.44-0.56	<.001
Sex		0.84	0.80-0.88	<.001
BMI	≤18.4	Reference		
	18.5-24.9	1.20	1.13-1.28	<.001
	25-29.9	1.22	1.13-1.33	<.001
	30-34.9	1.19	1.02-1.38	.026
	≥35	1.08	0.95-1.21	.239
	NA	0.97	0.86-1.09	.595
JCS at admission	0	Reference		
	1	0.81	0.78-0.85	<.001
	2	0.56	0.52-0.61	<.002
	3	0.39	0.34-0.45	<.003
CCI	0	Reference		
	1	0.91	0.86-0.96	.001
	2	0.92	0.86-0.97	.003
	≥3	0.86	0.81-0.92	<.001
Emergency transportation	0	Reference		
	1	1.14	1.09-1.19	<.001
	NA	0.58	0.19-1.76	.332
Hospital readmission		0.89	0.80-0.98	.021
Percutaneous endoscopic gastrostomy		0.37	0.28-0.48	<.001
Nasogastric tube		0.20	0.18-0.21	<.001
Tissue plasminogen activator		1.42	1.26-1.59	<.001
Edaravone		0.97	0.93-1.02	.067
Mechanical thrombectomy		1.69	1.48-1.92	<.001
Stroke care unit		1.03	0.96-1.09	.900
LOS (d)	≤14	Reference		
	15-30	1.03	0.97-1.08	.306
	31-44	0.99	0.93-1.06	.823
	45-60	1.08	0.99-1.18	.068
	≥61	1.41	1.32-1.52	<.001

NOTE. BMI calculated as weight in kilograms divided by height in meters squared.

Abbreviations: CI, confidence interval; NA, not applicable.

Table 3 shows multivariable logistic regression analysis using multiple imputation for the BI stratified by mRS score. The patients were stratified by mRS value to describe the relationship between rehabilitation and ADL. The same procedure in table 3 was used to adjust for all the variables. The effects of early rehabilitation and rehabilitation duration per day on ADL improvement were significantly related except for early rehabilitation in group mRS5. Early rehabilitation and rehabilitation duration per day showed different trends as mRS score increased. In terms of the duration effect of rehabilitation, the effectiveness of increased rehabilitation per day increased as the mRS score increased. In contrast, the effect of early rehabilitation on the improvement of ADL decreased as the mRS score increased, and an effect could not be demonstrated for the mRS5 group.

**Table 3:** Multivariable logistic regression analysis using multiple imputation for the Barthel Index stratified by mRS

	mRS3 (n=22,870)				
Variable		n	Odds	95% CI	*P* Value
Early rehabilitation	Usual	2172	Reference		
	Early	20,419	1.26	1.13-1.40	<.001
Duration of rehabilitation per day (h)	≥1	16,798	Reference		
	1.1-2.0	4818	1.35	1.25-1.46	<.001
	≥2.1	975	2.13	1.79-2.54	<.001

	mRS4 (n=22,686)				
Variables		N	Odds	95% CI	*P* Value

Early rehabilitation	Usual	2457	Reference		
	Early	19,961	1.17	1.06-1.30	.002
Duration of rehabilitation per day (h)	≥1	16,047	Reference		
	1.1-2.0	5104	1.32	1.23-1.42	<.001
	≥2.1	1267	2.63	2.27-3.05	<.001

	mRS5 (n=7967)				
Variable		N	Odds	95% CI	*P* Value

Early rehabilitation	Usual	1262	Reference		
	Early	6625	1.04	0.86-1.25	.689
Duration of rehabilitation per day (h)	≥1	5936	Reference		
	1.1-2.0	1438	1.47	1.25-1.73	<.001
	≥2.1	513	2.73	2.15-3.47	<.001

NOTE. We used the following as covariates: age, BMI, JCS at admission, CCI, emergency transportation, hospital readmission, percutaneous endoscopic gastrostomy, nasogastric tube, tissue plasminogen activator, edaravone, mechanical thrombectomy, stroke care unit, and length of hospital stay.

Abbreviation: CI, confidence interval.

Table 4 shows multivariable logistic regression analysis stratified using multiple imputation for very early rehabilitation. The association between very early rehabilitation and ADL is stratified by mRS score. Very early rehabilitation was just as effective as early rehabilitation, but no association was found for the mRS5 group (mRS3, *P*≤.001; mRS4, *P*=.002; mRS5, *P*=.689).

**Table 4:** Multivariable logistic regression analysis stratified using multiple imputations for very early rehabilitation

Variable		n	Odds	95% CI	*P* Value
Very early rehabilitation	mRS3,4.5	35,484	1.11	1.05-1.17	<.001
	mRS3	15,284	1.15	1.07-1.24	<.001
	mRS4	15,212	1.17	1.06-1.30	.002
	mRS5	4988	1.04	0.86-1.25	.689

NOTE. We used the following as covariates: age, BMI, JCS at admission, CCI, emergency transportation, hospital readmission, percutaneous endoscopic gastrostomy, nasogastric tube, tissue plasminogen activator, edaravone, mechanical thrombectomy, stroke care unit, and length of hospital stay. The reference for all analyses was the usual rehabilitation.

Abbreviation: CI, confidence interval.

## Discussion

This study was the first to reveal the effectiveness of acute stroke rehabilitation in patients who required assistance before hospitalization. In the early rehabilitation and very early rehabilitation group, the improvement in ADL from admission to discharge was better than in the usual rehabilitation group for mRS3 and mRS4 patients. Regarding the duration of rehabilitation per day, the degree of improvement in ADL was higher for those who received >1 hour of rehabilitation than for those with <1 hour of rehabilitation. The present results are generally consistent with those from previous observational and intervention studies.^7^^,^^22^^,^^23^ Rehabilitation of patients with acute stroke who needed assistance before admission was associated with improvement of ADL; specifically, both early rehabilitation and an increased duration of rehabilitation per day were effective at improving ADL.

In our study population, the highest incidence of stroke was in patients aged 80-89 years. Compared with epidemiologic data on stroke in Japan,^24^ our study population was slightly older and the proportion of women was higher than the proportion of men, a reverse trend to epidemiologic data. It was possible that this study's findings were influenced by participants in a higher age group than the epidemiologic data on cerebral infarction in Japan.

Early rehabilitation of patients with stroke who required assistance prior to admission was as effective in improving ADL as in patients with stroke who did not require assistance before admission.^25^ In previous studies examining the prognostic factors for the outcomes in older patients with ischemic stroke, frailty status, such as reduced walking speed and grip strength before stroke onset, was associated with a poor prognosis after stroke onset.^26^ Patients with cerebral infarction who required assistance before the onset of stroke were thought to be less likely to benefit from early rehabilitation in terms of improving ADL. However, this study revealed that early rehabilitation of patients with stroke who required assistance before admission was associated with improvement of ADL.

We found that very early rehabilitation was also effective in patients with stroke who required assistance before the onset of stroke. This result differed from the results of a previous study that investigated very early rehabilitation of patients with stroke who did not require assistance before the onset of stroke.^27^ A Very Early Rehabilitation Trial, a large randomized controlled study of the effects of very early rehabilitation within 24 hours of the onset, found that very early intervention did not have a positive effect on ADL outcomes.^28^ Previous studies included special care unit inpatients, and the results were obtained in a different setting from this study. This study included inpatients admitted to acute care hospitals, including special care unit inpatients. It was possible that patients in the earlier study were hospitalized earlier from the onset and had a more severe stroke than those in the present study. The indication criteria for treatment with tPA is defined as 4.5 hours from stroke onset.^29^ In the previous study, the percentage of tPA use was 23% in the very early rehabilitation group (present study, 4.8%). This study may have benefited from very early rehabilitation because of the difference in the time from stroke onset to hospitalization compared with the previous studies. Herein, we found that very early rehabilitation was associated with improved ADL in patients who required assistance before the onset of stroke.

This study showed that increasing the average rehabilitation duration per day for patients who required assistance before the onset of stroke led to improved ADL, similar to previous studies. Increasing the duration of rehabilitation after stroke reportedly affects physical function at discharge and ADL improvement after 3 months.^30^, ^31^, ^32^ In a study examining the rehabilitation duration of patients with stroke, ADL improved when the rehabilitation duration was ≥2 hours compared with 1 hour and 1-2 hours, similar to the results of this study.^33^ Our study revealed that a longer duration of rehabilitation can be applied to patients who required assistance before hospitalization and who were considered to have low physical functions.

In the analysis of the effects of rehabilitation on patients stratified by prehospital mRS, score early and very early rehabilitation did not result in ADL improvement in patients with an mRS score of 5 before hospital admission. In contrast, the duration of rehabilitation per day was associated with ADL improvement in all mRS strata. Previous studies on early rehabilitation are difficult to compare with this study because the participants had an mRS score ≥2 before admission. Herein, we found that the effect of early rehabilitation on the improvement of ADL differed depending on the care required before hospitalization. This suggests that it is necessary to consider long-term physical function in early rehabilitation intervention.

### Study limitations

This study has several limitations. First, it used medical administrative databases, which do not represent nearly half of patients in Japan. Second, the primary outcome had 20.8% missing data of the total population. We performed the analysis using multiple imputation for this missing data, which may be insufficient to control bias. However, the basic characteristics of the groups with and without missing values were balanced (0.1<standardized mean difference) except for the body mass index and emergency transport variables. In the analysis of rehabilitation for ADL improvement calculated with missing values, early rehabilitation, very early rehabilitation, and daily duration of rehabilitation each had the same direction of results as when missing values were imputed (supplemental tables S5-7).

Third, this study does not allow for discussions on the low-volume, high-frequency interventions of early rehabilitation discussed in A Very Early Rehabilitation Trial, because the database contains data on total rehabilitation hours per day; however, it does not contain information on how many interventions are performed in a day.^28^

Fourth, lack of information on the site of stroke onset and the location of the infarction plus lack of background factors for nutritional status may have resulted in insufficient adjustment for background factors and severity of the disease. Fifth, this study could not examine the long-term outcomes of rehabilitation because the data were obtained during hospitalization in an acute care hospital. The transition from acute hospitalization to a rehabilitation hospital and outpatient treatment differs from country to country. In Japan, the policy is to shorten the length of acute hospitalization and to transfer patients to rehabilitation hospitals as early as possible. This study focuses on the period from admission to discharge from acute care hospitals. The effect after transfer to a rehabilitation hospital is not clarified.

## Conclusions

Early implementation of rehabilitation and a longer duration of rehabilitation per day improved ADL for patients who required assistance before the onset of cerebral infarction. However, the indications for early rehabilitation need to be considered according to background factors such as the patient's physical status before admission. More information is needed from randomized trials to confirm the effects of early rehabilitation and the daily duration of rehabilitation on ADL recovery in patients with stroke who require preadmission assistance.

## Supplier

a. R statistical software version 4.2.0; R Core Team.

## Appendix Supplementary materials

Image, application 1
