# Supplementary material for: Rehabilitation of Patients With Acute Ischemic Stroke Who Required Assistance Before Hospitalization Contributes to Improvement in Activities of Daily Living: A Nationwide Database Cohort Study
Source: Arch Rehabil Res Clin Transl. 2022 Aug 5;4(4):100224. doi: 10.1016/j.arrct.2022.100224 (PMC9761257; doi:10.1016/j.arrct.2022.100224)
Supplement: Supplementary file 1 [file mmc1.docx]

**SUPPLEMENTARY MATERIAL**

**ARTICLE TITLE: Rehabilitation of patients with acute ischemic stroke who required assistance before hospitalization contributes to improvement in activities-of-daily-living: A nationwide database cohort study**

| Variables | N | % |
| --- | --- | --- |
| Total missing values | 12873 | 24.1% |
| BI | 11169 | 20.8% |
| BMI | 2398 | 4.5% |
| Emergency transportation, | 22 | 0.04% |

Table S1. Number and percentage of missing values

BI: Barthel index, BMI: body mass index

Table S2. Outcomes for each rehabilitation group

| Rehabilitation |  | n(%) | | Change in BI, mean (SD) | | p |
| --- | --- | --- | --- | --- | --- | --- |
| VER within 1day |  | 35484 | (66.4) | 2.4 | (5.5) | <0.001 |
| usual rehabilitation (rehabilitation started after 2 days) |  | 18039 | (33.7) | 2.1 | (6.0) |  |
| ER within 3days |  | 47082 | (88.0) | 2.4 | (5.6) | <0.001 |
| usual rehabilitation (rehabilitation started after 4 days) |  | 6441 | (12.0) | 1.7 | (6.1) |  |
| Duration of rehabilitation per day, hour | ≤1 | 38781 | (73.3) | 1.9 | (5.7) | <0.001 |
|  | 1.1–2.0 | 11360 | (21.4) | 3.0 | (5.6) |  |
|  | ≥2.1 | 2755 | (5.2) | 4.6 | (5.0) |  |

BI: Barthel index, ER: early rehabilitation; IQR: interquartile range, LOS: length of hospital stay, VER: very early rehabilitation

Table S3. Outcomes for each rehabilitation group

| Rehabilitation |  | LOS, median [IQR] | | p |
| --- | --- | --- | --- | --- |
| VER within 1day |  | 25 | [15,46] | <0.001 |
| usual rehabilitation (rehabilitation started after 2 days) |  | 28 | [17,47] |  |
| ER within 3days |  | 25 | [16,45] | <0.001 |
| usual rehabilitation (rehabilitation started after 4 days) |  | 34 | [21,58] |  |
| Duration of rehabilitation per day, hour | ≤1 | 24 | [15,41] | <0.001 |
|  | 1.1–2.0 | 30 | [18,55] |  |
|  | ≥2.1 | 71 | [38,109] |  |

BI: Barthel index, ER: early rehabilitation; IQR: interquartile range, LOS: length of hospital stay,

VER: very early rehabilitation

Table S4. Outcomes for each rehabilitation group

|  |  | Discharge destination, n (%) | | | | | | | | |
| --- | --- | --- | --- | --- | --- | --- | --- | --- | --- | --- |
| Rehabilitation |  | Home | | Facility | | Transfer | | Other | | p |
| VER within 1day |  | 12,786 | (36.1) | 7,815 | (22.0) | 14,823 | (41.8) | 29 | (0.1) | <0.001 |
| usual rehabilitation (rehabilitation started after 2 days) |  | 5,686 | (31.6) | 3,593 | (19.9) | 8,722 | (48.4) | 21 | (0.1) |  |
| ER within 3days |  | 16,449 | (35.0) | 10,123 | (21.5) | 20,429 | (43.4) | 42 | (0.1) | <0.001 |
| usual rehabilitation (rehabilitation started after 4 days) |  | 2,023 | (31.5) | 1,285 | (20.0) | 3,116 | (48.4) | 8 | (0.1) |  |
| Duration of rehabilitation per day, hour | ≤1 | 12,420 | (32.1) | 7,933 | (20.5) | 18,354 | (47.4) | 41 | (0.1) | <0.001 |
|  | 1.1–2.0 | 4,208 | (37.1) | 2,658 | (23.4) | 4,474 | (39.4) | 7 | (0.1) |  |
|  | ≥2.1 | 1,579 | (57.4) | 648 | (23.5) | 524 | (19.0) | 2 | (0.1) |  |

BI: Barthel index, ER: early rehabilitation; IQR: interquartile range, LOS: length of hospital stay, VER: very early rehabilitation

| Characteristics | | The group with no missing values | | The group with missing values | |  |
| --- | --- | --- | --- | --- | --- | --- |
|  |  | N = 40650 | | N=12873 | | SMD* |
| Age, years, n (%) | ≤59 | 1362 | (3.4) | 492 | (3.8) | 0.056 |
|  | 60‐69 | 3412 | (8.4) | 1159 | (9.0) |  |
|  | 70-79 | 9139 | (22.5) | 3035 | (23.6) |  |
|  | 80-89 | 17387 | (42.8) | 5465 | (42.5) |  |
|  | ≥90 | 9350 | (23.0) | 2722 | (21.1) |  |
| Sex (female), n (%) | | 22521 | (55.4) | 6841 | (53.1) | 0.045 |
| BMI, n (%) | ≤18.5 | 7969 | (19.6) | 1802 | (14.0) | 0.682 |
|  | 18.5-24.9 | 24401 | (60.0) | 6297 | (48.9) |  |
|  | 25-29.9 | 5825 | (14.3) | 1613 | (12.5) |  |
|  | 30-34.9 | 820 | (2.0) | 240 | (1.9) |  |
|  | ≥35 | 1635 | (4.0) | 523 | (4.1) |  |
|  | NA | 0 | 0.0 | 2398 | (18.6) |  |
| CCI, n (%) | 0 | 12564 | (30.9) | 4103 | (31.9) | 0.025 |
|  | 1 | 13591 | (33.4) | 4315 | (33.5) |  |
|  | 2 | 8575 | (21.1) | 2642 | (20.5) |  |
|  | ≥3 | 5920 | (14.6) | 1813 | (14.1) |  |
| mRS | 3 | 16976 | (41.8) | 5894 | (45.8) | 0.081 |
|  | 4 | 17437 | (42.9) | 5249 | (40.8) | 0.043 |
|  | 5 | 6237 | (15.3) | 1730 | (13.4) | 0.054 |
| JCS at admission, n (%) | 0 | 12835 | (31.6) | 4092 | (31.8) | 0.024 |
|  | 1 | 20527 | (50.5) | 6582 | (51.1) |  |
|  | 2 | 5184 | (12.8) | 1584 | (12.3) |  |
|  | 3 | 2104 | (5.2) | 615 | (4.8) |  |
| Emergency transportation, n (%) | 0 | 19074 | (46.9) | 5412 | (42.0) | 0.113 |
|  | 1 | 21576 | (53.1) | 7439 | (57.8) |  |
|  | NA | 0 | 0.0 | 22 | (0.2) |  |
| Hospital readmission, n (%) | | 2177 | (5.4) | 601 | (4.7) | 0.031 |
| Percutaneous endoscopic gastrostomy, n (%) | | 1032 | (2.5) | 239 | (1.9) | 0.047 |
| Nasogastric tube, n (%) | | 8346 | (20.5) | 2477 | (19.2) | 0.032 |
| Edaravone, n (%) | | 20223 | (49.7) | 6628 | (51.5) | 0.035 |
| Tissue plasminogen activator, n (%) | | 1921 | (4.7) | 661 | (5.1) | 0.019 |
| Mechanical thrombectomy, n (%) | | 1351 | (3.3) | 445 | 0.007 | 0.031 |
| Stroke care unit, n (%) | | 5968 | (14.7) | 1727 | 0.036 | 0.047 |

Table S5. Basic characteristics of groups with missing values vs groups with no missing values

*SMD<0.1 indicates a good balance.
MI： body mass index, CCI: Carlson comorbidity index, JCS: Japan Coma Scale, LOS: length of hospital stay; mRS: modified Rankin Scale; SMD: Standardised mean difference; NA: not applicable

Table S6. Relationship between rehabilitation and ADL improvement using multivariable logistic regression analysis with complete case analysis

| Variables | | Odds ratio | 95% CI | | p-value |
| --- | --- | --- | --- | --- | --- |
| Early rehabilitation | Usual | Reference | | |  |
|  | Early | 1.21 | (1.13-1.30) | | <0.001 |
| Duration of rehabilitation per day, hour | ≤1 | Reference | |  |  |
|  | 1.1–2.0 | 1.45 | (1.38-1.53) | | <0.001 |
|  | ≥3.1 | 2.67 | (2.42-2.96) | | <0.001 |
| Age, years | ≤59 | Reference | |  |  |
|  | 60‐69 | 0.89 | (0.78-1.02) | | 0.1 |
|  | 70-79 | 0.77 | (0.68-0.87) | | <0.001 |
|  | 80-89 | 0.61 | (0.54-0.68) | | <0.001 |
|  | ≥90 | 0.44 | (0.39-0.51) | | <0.001 |
| Sex |  | 0.85 | (0.82-0.90) | | <0.001 |
| BMI, kg/m^2^ | ≤18.4 | Reference | |  |  |
|  | 18.5-24.9 | 1.29 | (1.22-1.37) | | <0.001 |
|  | 25-29.9 | 1.34 | (1.24-1.45) | | <0.001 |
|  | 30-34.9 | 1.31 | (1.12-1.54) | | 0.026 |
|  | ≥35 | 1.11 | (0.98-1.25) | | 0.1 |
| JCS at admission | 0 | Reference | |  |  |
|  | 1 | 0.82 | (0.78-0.86) | | <0.001 |
|  | 2 | 0.51 | (0.47-0.56) | | <0.001 |
|  | 3 | 0.34 | (0.29-0.39) | | <0.001 |
| CCI | 0 | Reference | |  |  |
|  | 1 | 0.89 | (0.85-0.94) | | <0.001 |
|  | 2 | 0.92 | (0.85-0.96) | | 0.002 |
|  | ≥3 | 0.86 | (0.80-0.92) | | <0.001 |
| Emergency transportation | 0 | Reference | |  |  |
|  | 1 | 1.20 | (1.14-1.25) | | <0.001 |
| Hospital readmission |  | 0.86 | (0.78-0.94) | | 0.002 |
| Percutaneous endoscopic gastrostomy |  | 0.31 | (0.24-0.40) | | <0.001 |
| Nasogastric tube |  | 0.18 | (0.17-0.20) | | <0.001 |
| Tissue plasminogen activator |  | 1.37 | (1.22-1.53) | | <0.001 |
| Edaravone |  | 0.99 | (0.95-1.04) | | 0.8 |
| Mechanical thrombectomy |  | 1.72 | (1.50-1.97) | | <0.001 |
| Stroke care unit |  | 1.03 | (0.96-1.09) | | 0.4 |
| LOS, days | ≤14 | Reference | |  |  |
|  | 15-30 | 1.06 | (1.00-1.12) | | 0.066 |
|  | 31-44 | 1.01 | (0.94-1.08) | | 0.8 |
|  | 45-60 | 1.12 | (1.03-1.23) | | 0.01 |
|  | ≥61 | 1.53 | (1.42-1.65) | | <0.001 |

BMI: body mass index, CCI: Charlson comorbidity index, CI: confidence interval, JCS: Japan Coma Scale, LOS: length of hospital stay, NA, not applicable

Table S7. Multivariable logistic regression analysis stratified with complete case analysis for very early rehabilitation

| Variables |  | Odds | 95% CI | p-value |
| --- | --- | --- | --- | --- |
| Very early rehabilitation | Usual | Reference |  |  |
|  | Very early | 1.10 | (1.05-1.16) | <0.001 |

We used the following as covariates: age, BMI, JCS at admission, CCI, emergency transportation, hospital readmission, percutaneous endoscopic gastrostomy, nasogastric tube, tissue plasminogen activator, edaravone, mechanical thrombectomy, stroke care unit, and length of hospital stay

BMI: body mass index, CCI: Charlson comorbidity index, CI: confidence interval, JCS: Japan Coma Scale, mRS: modified Rankin Scale
